# The Association of *APOE ε*4 Allele with Retinal Layer Thickness and Microvasculature in Older Adults: Optic Nerve Decline and Cognitive Change Study

**DOI:** 10.3390/jcm12196219

**Published:** 2023-09-27

**Authors:** Samran Sheriff, Ting Shen, Danit Saks, Angela Schultz, Heather Francis, Wei Wen, Jiyang Jiang, Mehdi Mirzaei, Veer Gupta, Maria Fiatarone Singh, Perminder S. Sachdev, Stuart L. Graham, Vivek Gupta

**Affiliations:** 1 Macquarie Medical School, Macquarie University, Sydney, NSW 2109, Australia; 2 Department of Ophthalmology, Shanghai General Hospital (Shanghai First People’s Hospital), School of Medicine, Shanghai Jiao Tong University, Shanghai 200240, China; ting.shen@mq.edu.au; 3 School of Psychological Sciences, Macquarie University, Sydney, NSW 2019, Australia; 4 Neurology Department, Royal North Shore Hospital, Sydney, NSW 2065, Australia; 5 Centre for Healthy Brain Ageing, University of New South Wales, Sydney, NSW 2052, Australia; 6 Neuropsychiatric Institute, Prince of Wales Hospital, Sydney, NSW 2031, Australia; 7 School of Medicine, Deakin University, Melbourne, VIC 3125, Australia; 8 Sydney Medical School, Faculty of Medicine and Health, The University of Sydney, Sydney, NSW 2050, Australia; 9 Hinda and Arthur Marcus Institute for Aging Research, Hebrew SeniorLife, Boston, MA 02131, USA

**Keywords:** APOE, OCT, dementia, MRI, retina

## Abstract

Purpose: To investigate the relationship between the apolipoprotein E (APOE) ε4 allele and retinal structural and vascular characteristics in older adult participants from several research studies. We also studied the relationship between these structural and vascular characteristics with multifocal visual evoked potential (mfVEP) indices, neuropsychological parameters and MRI brain volumes in these participants. Methods: In this study, 109 participants with a mean (SD) age of 67.1 (9.0) years were recruited. Participants were classified as APOE ε4 carriers or non-carriers based on the presence or absence of the ε4 allele. Baseline measurements included peripapillary retinal nerve fibre layer optical coherence tomography (RNFL OCT), and OCT–angiography (OCT-A) for evaluation of the retinal layer thickness and vessel density (VD) parameters. A multifocal visual evoked potential (mfVEP) test, including amplitude and latency, was used to assess the visual pathway function. Finally, cognitive function was evaluated using a battery of neuropsychological tests. OCT-A images were analysed in ImageJ to quantify VD in the superficial and deep vascular plexus and the size of the foveal avascular zone (FAZ). The relationship between carriers of APOE ε4 allele and these ocular parameters was analysed using generalised estimating equation (GEE) models and data adjusted for age, sex and inter-eye differences as within-subject variables (*p* < 0.05). Results: Twenty-four participants were APOE ε4 carriers. Temporal RNFL thickness was decreased in APOE ε4 carriers (*p* < 0.01). Vessel density between carriers and non-carriers was not significantly different at either the superficial or deep level. The FAZ area was significantly smaller in ε4 carriers in both superficial (*p* < 0.01) and deep layers (*p* < 0.003). Conclusions: Retinal abnormalities were present in participants with increased genetic risk of dementia due to presence of the ε4 allele. These findings provide preliminary evidence for their potential role in the diagnosis of dementia.

## 1. Introduction

Dementia and cognitive decline due to aging continue to be a major public health burden worldwide. The current prevalence of dementia among those aged over 60 is 5–7%, with the number of individuals diagnosed with dementia likely to double as a result of the aging population if current trends continue [1]. Cognitive decline remains a key feature of neurodegenerative diseases and is often a major complaint of participants with disorders such as Alzheimer’s disease (AD). Early and definitive diagnosis of AD remains a key challenge and the subject of current research. With the development of disease-modifying treatments, there is a need for early and definitive diagnosis, which may rely on clinical biomarkers. Currently available biomarkers for AD include positron emission tomography (PET) imaging and cerebral spinal fluid (CSF) examination. Whilst useful, both are highly expensive, not readily available, and invasive. This makes them unsuitable for large-scale population screening.

The retina is often considered a window to the brain and offers an opportunity to potentially function as a biomarker. The retina is physically connected to the brain via axons that form the optic nerve, which allow both anterograde and retrograde forms of communication between the two structures. The close relationship between the brain and retina, including their anatomical substructures, provides a form of non-invasive imaging that can allow for a better understanding of processes that occur in both organs, for both AD and healthy aging.

An individual’s genetic predisposition may also serve as a biomarker for the development of AD. A familial history of AD increases the risk of developing the condition, as does having at least one copy of the apolipoprotein E (APOE) *ε*4 allele. Pathological features of AD are the deposition of amyloid β (Aβ) peptide and hyperphosphorylation of tau protein; however, recently, vascular factors have also been considered to be involved in the pathophysiology of AD [2]. The APOE *ε*4 allele is one of the strongest genetic risk factors for sporadic forms of AD. The *ε*4 allele is estimated to be expressed in more than 50% of AD patients. Having one copy of the *ε*4 allele increases the risk of developing AD two- to threefold, whilst being homozygous for *ε*4 increases the risk tenfold. [3]. Apolipoprotein E regulates cholesterol metabolism and modulates the clearance of amyloid β. The precise biological mechanisms linking the APOE genotype and AD, however, are not well understood. Cross-sectional studies in older adults without dementia have suggested that APOE ε4 is associated with impairment in memory, executive function, processing speed and global cognitive function [4,5]. Additionally, neuroimaging studies have utilised PET scanning to investigate the involvement of the APOE ε2 allele in episodic memory decline and AD disease progression [4,5].

An individual’s APOE *ε*4 carrier status could thus be used as a marker of increased risk of the development of AD in an aging cohort and to examine relationships with potential biomarkers in the eye. Although not currently used clinically for AD progression or diagnosis, retinal examination may offer valuable insights into understanding the complex nature of this condition. Due to the similarities between the retinal and cerebral vasculature, which share embryological origin as well as physiological and anatomical properties, it is logical to use the retinal vascular network to understand the characteristics of vascular cerebral pathologies such as AD [2,6,7]. MRI studies have revealed notable differences in cerebral microvasculature and vascular features between APOE *ε*4-positive and *ε*4-negative individuals. Additionally, APOE *ε*4-mediated neurodegeneration is likely related to vascular pathology; however, the precise mechanism through which these APOE *ε*4-related vascular changes contribute to AD pathology remains unclear [8,9].

Ocular coherence tomography–angiography (OCT-A) is a recent extension of traditional OCT imaging that allows, without contrast injection, the acquisition of images that can be processed and analysed to assess features such as size, shape, vascular blood flow and density [10]. This technique has been used to study the microvasculature changes in AD patients, such as loss of vascular density (VD) and increase in foveal avascular zone (FAZ) compared to individuals with mild cognitive impairment (MCI) and healthy control individuals [11]. Vascular changes have also been identified in preclinical patients with AD; however, little is known about the relationship between changes in the retinal microvasculature and retinal neuronal layers with the *APOE-ε*4 genotype [12,13,14]. The results from these previous studies suggest that it would be beneficial to investigate cognitively healthy individuals who have a genetic predisposition to developing AD, as changes related to neurodegeneration appear decades before the onset of clinical symptoms and the structural changes identified early may be useful diagnostically.

It has been shown that the retinal changes measured by OCT–angiography (OCTA) could be biomarkers of AD. Diagnosis in early stages before irreversible AD neurological damage takes place is important for the development of new therapeutic interventions [9,15].

Therefore, in the present study, we aimed to examine the effects of the APOE ε4 genotype and its relationship to various cognitive domains, ophthalmic parameters and brain volumes in a cohort of community-dwelling aging adults. We aimed to investigate specifically whether the APOE ε4 allele is associated with OCT-A, multifocal visual evoked potential (mfVEP) and/or brain magnetic resonance imaging (MRI) parameters.

## 2. Methods

### 2.1. Participant Recruitment

Between July 2020 and August 2021, participants were enrolled from the general community for participation in the Optic Nerve Decline and Cognitive Change (ONDCC) study [16]. This cross-sectional investigation of aging participants was conducted to examine the relationships between genetic profile and cognitive and ophthalmic parameters.

The inclusion criteria included: (1) male or female, aged ≥ 50 years; (2) ability to provide consent for study participation, either by themselves or through a reliable informant; (3) ability to speak and write English sufficiently well to give written informed consent, complete self-reported questionnaires, and undergo psychometric assessment. The control group had no history of head trauma or neurological or psychiatric illness.

Exclusion criteria were participants with a history of macular degeneration or other retinal disorders, a best-corrected visual acuity of <6/9, or any other ocular condition that in the opinion of the investigators would represent a complicating factor. One such complicating factor would be the exclusion of participants with pre-perimetric glaucoma. As visual field testing was not conducted in this study, participants suspected of having characteristic glaucomatous changes in the optic disc as seen on OCT imaging were excluded. Those who had had ocular surgery in the previous two months or were unable to adequately understand the procedures of the study were also excluded. Any evidence of dementia, depression, neurotrauma, progressive malignancy, or any other major neurological disease. Demographic information, including age, sex and education, were collected at the time of recruitment. All participants provided written informed consent.

This study was conducted as per the study protocols approved by the Macquarie University Human Research and Ethics Committee (52019553810900), with all aspects of the study adhering to the tenets of the Declaration of Helsinki.

### 2.2. Ophthalmic Examination and Optical Coherence Tomography (OCT)

**Figure 1:**
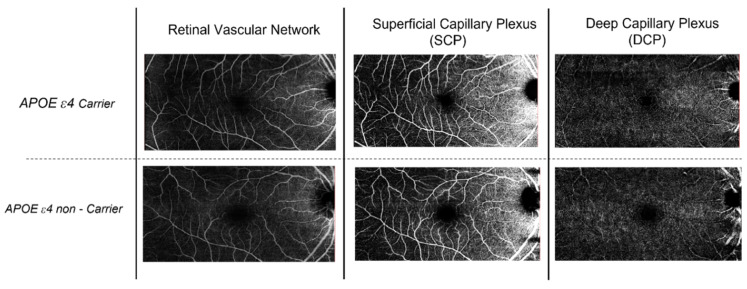
Single-subject comparison of optical coherence tomography–angiography (OCT-A) scans in a carrier versus non-carrier of similar age.

**Figure 2:**
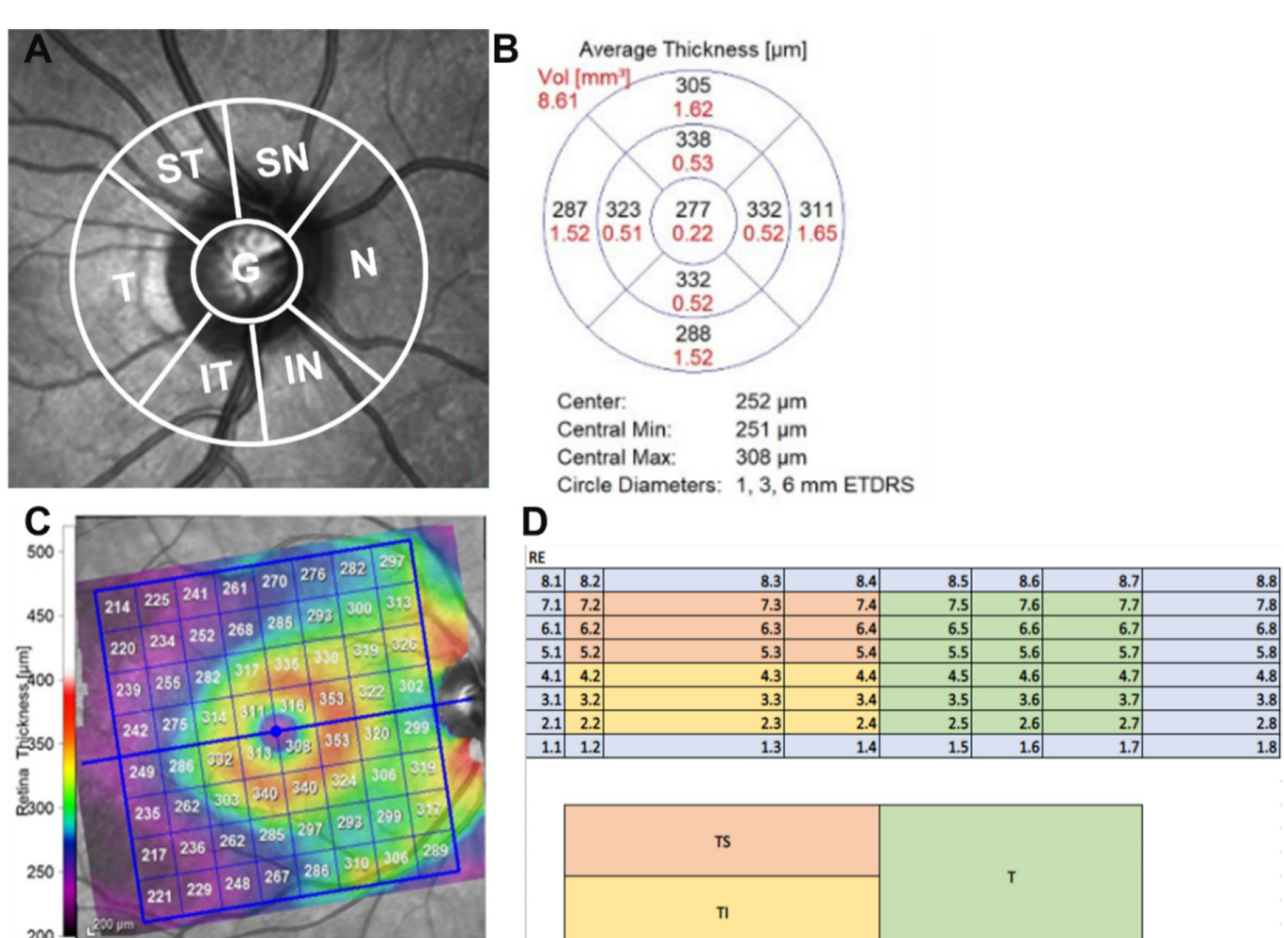
OCT data of retinal nerve fibre layer thickness analysis (RNFL). (**A**) Peripapillary retinal nerve fibre layer (RNFL) sectors used for analysis. Sectors include supero–temporal (st), supero–nasal (SN), Nasal (N), infero-nasal (IN), infero-temporal (IT), temporal (T), and global (G)). (**B**) Diagrammatic output showing concentric rings and quadrants for analysis of macular RNFL thickness provided by Heidelberg OCT. (**C**) Posterior pole retina thickness map (8 × 8 grid) Heidelberg OCT output displays the retinal thickness of the entire posterior pole (30 degrees × 25 degrees OCT volumetric scan); compressed colour scale used to localise the differences in retinal thickness. For each cell of the grid, the mean retinal thickness is given (um). (**D**) Manual calculation of posterior pole (temporal, temporal superior and temporal inferior) on 8 × 8 grid.

A detailed ophthalmic examination was conducted in all participants, including corrected distance visual acuity (logarithm of the minimum angle of resolution; (logMAR)) and intraocular pressure (mmHg). Detailed anterior segment and fundus examinations were performed, and wide-field colour fundus images were taken using a laser scanning ophthalmoscope (Optomap; Optos Plc., Dunfermline, UK). Examination of the microvasculature and retinal thickness of each layer of the macula was performed using a Spectralis OCT (Spectralis HRA + OCT; Heidelberg Engineering, Heidelberg, Germany). All participants underwent three OCT scans on the same day as their cognitive evaluation. A peripapillary ring (SD-OCT RNFL diameter, 3.50 mm) and posterior-pole scan were conducted. All images fulfilled the spell-out OSCAR-IB criteria [17], with an enhanced automatic real-time functioning (ART) level of 25. Spectralis OCT software (Spectralis HRA + OCT; Heidelberg Engineering, Heidelberg, Germany, Version 4.0) allowed for automatic segmentation with options for manual adjustment and identification of the upper and lower RNFL limits to minimize artefacts such as defocus, shadows, eye movement and blinking, and calculate the average RNFL thickness. A Heidelberg Spectralis Axonal exam report was generated and viewed following each participant’s scan. Peripapillary RNFL thickness global retinal nerve fibre layer (gRNFL), as well as sectoral RNFL thicknesses, including nasal, temporal, temporal inferior, temporal superior, nasal inferior, and nasal superior quadrants, were measured for analysis in this study. Non-invasive vascular imaging was conducted using the Spectralis HRA + OCT OCT-A module to examine the retinal microvasculature covering a macular area of 6 × 6 mm cantered on the fovea for the superficial capillary plexus (SCP) and deep capillary plexus (DCP) of the retina. As Spectralis does not automate density values, vessel density analysis was performed using ImageJ software 1.53o (National Institutes of Health, Bethesda, MD, USA) with a method adapted from Elfarnawany et al. [10,18]. The macula OCTA image was imported into ImageJ software, 1.53o where it was binarised and skeletonised. FAZ features were measured with increased magnification. The border of the FAZ was manually traced using the polygon selection tool. Measurable features including area (mm^2^), perimeter (mm), and circularity were defined using the set measurements of shape descriptor features within the software 1.53o. Circularity is defined as an index such that closer to 1 indicates more circular in shape and closer to 0 indicates greater irregularity. The projection artefacts of the superficial layer were removed in the deep-layer images using built-in software 1.53o native to the Spectralis HRA + OCT. All images were exported into the ImageJ 1.50 software to measure the FAZ area and VD. We manually outlined the FAZ using the polygon selection tool and calculated the VD of the 6 × 6 mm macula, except the FAZ (central foveal 0.5 mm radius area). The OCT-A data were collected and evaluated independently by two researchers, and the average values used for the statistical evaluation. The analysis of the FAZ and VD in ImageJ were conducted blindly to avoid potential bias by researchers who were masked to the APOE genotype and all clinical measures and had no knowledge of the results of MRI imaging. The manual scans were also reviewed by two researchers before exporting from the instrument. Eyes with retinal diseases (e.g., age-related macular degeneration, diabetic retinopathy, epiretinal membrane, and macular hole), optic nerve diseases (e.g., glaucoma, and ischemic optic neuropathy), and significant media opacity with poor quality (signal strength < 70 on OCTA and  <25 on OCT) were excluded from the analyses (Figure 1 and Figure 2).

Retinal capillary density maps derived from OCTA images for an apolipoprotein E (APOE) ε4 carrier and non-carrier. The first column shows raw OCTA images where white pixels represent retinal vessels within a 3 × 3 mm^2^ region surrounding the naturally occurring foveal avascular zone of the retina. The second and third columns represent the superficial capillary plexus. (SCP) and deep capillary plexus (DCP).

### 2.3. Neuropsychological Evaluation

All study participants had a detailed neuropsychological evaluation using a battery of tests performed by trained personnel before OCT data acquisition. Initially, the premorbid predicted full-scale IQ was calculated using the following equation: 126.41–0.9775 * (NART) errors (National Adult Reading Test) [19]. The cognitive battery assessed various cognitive domains, including verbal episodic memory: California Verbal Learning Test second edition (CVLT-II) [20], logical memory (immediate, delay, recognition) [21], verbal fluency—Controlled Oral Word Association Task (COWAT) [22], processing speed—Symbol Digit Modalities Test (SDMT) [23], Trail-Making Test A [24], basic attention—digit span (forwards) [25], working memory—digit span (backwards, sequencing, total) [26], executive function—Trail-Making Test B, language—30-item Boston Naming Test (BNT) [27], and visuo-constructional ability—Rey Complex Figure Test (RCFT) [28]. Additionally, we conducted the Mini-Mental State Examination (MMSE) [29] as a screener of global cognitive function and the Geriatric Depression Scale (GDS) [30] as a depression screening tool.

### 2.4. Multifocal Visual Evoked Potential (mfVEP)

Multifocal visually evoked potential testing was performed utilising Vision Search 1 perimetry (VisionSearch, Sydney, Australia) controlled by Terra software 1.0 (VisionSearch, Sydney, Australia), and standard stimulus condition [31]. Fifty-eight closely packed segments (eccentricity up to 24 degrees) in a cortically scaled dartboard configuration were used. Four gold-disc electrodes (Grass, West Warwick, RI, USA) were used for bipolar recording with 2 electrodes positioned 4 cm apart on either side of the inion and 1 electrode placed 2.5 cm above and another 4.5 cm below the inion along the midline. Electrodes were positioned in a premade plastic casing for accurate positioning. Electrical signals were recorded by 2 channels, enabling monitoring of the difference between superior and inferior, as well as between left and right electrodes. The largest peak-to-trough amplitude within the interval of 70 to 210 ms was ascertained for each channel. The locus of maximum amplitude among the channel traces was selected automatically by Terra software 1.0 (VisionSearch), to generate a combined topographic map for amplitude and latency analysis. Traces obtained from the combined channel were selected for all tests for every individual segment of the visual field using a specially designed algorithm embedded in the instrument.

### 2.5. Magnetic Resonance Imaging (MRI) of the Brain

All MRI scans were acquired on a 3.0T GE Discovery^TM^ MR750w Wide Bore MRI scanner (GE Healthcare, Milwaukee, WI, USA) with a 32-channel Nova Head Coil and a software version of DV26.0_R01_1725.a, located at Macquarie Medical Imaging, NSW, Australia. The T1-weighted scans were acquired with a magnetisation-prepared rapid acquisition gradient echo (MPRAGE) pulse sequence with prospective motion correction (PROMO). The following scanning parameters were used: repetition time (TR) = 8.388 ms, echo time (TE) = 3.168 ms, inversion time (TI) = 900 ms, flip angle (FA) = 8°, pixel bandwidth = 244.141 Hz, and acquisition matrix = 256 × 256, 198 slices, yielding 1 mm isotropic voxels. Auto-calibrating reconstruction for Cartesian imaging (ARC) was applied for parallel imaging (acceleration factor = 3 in the phase-encoding direction).

### 2.6. Quantification of Brain Volumetric Measures and Quality Control

We conducted whole-brain volumetric analysis using T1-weighted MRAGE scans that were processed with standard FreeSurfer pipeline (version 7.1.0, Laboratories for Computational Neuroimaging, Charlestown, MA, USA). The assessment included volumetric analyses of the following brain regions: estimated total intracranial volume (ETIV), total grey matter (GM), cortex, cerebral white matter, cingulate grey matter, parietal grey matter, entorhinal cortex, third ventricle, fourth ventricle, thalamus, putamen, and hippocampus. Briefly, the processing included signal from the non-brain tissue, transforming to standard space, segmentation of subcortical white matter and deep grey matter structures, intensity normalisation, tessellation of the boundary between grey and white matter, automated topology correction, and reconstruction of grey-white and grey-cerebrospinal fluid surfaces. Volumes of cortical and subcortical structures were then quantified.

We then followed Enhancing Neuro Imaging Genetics through meta-Analysis (ENIGMA) Cortical Quality Control Protocol 2.0 to assure quality for the FreeSurfer results. Both internal and external quality control images were also checked visually. The unsatisfactorily segmented region volumes were excluded from further analyses. The number of removed values for each measure ranged from 0 to 15. Left banks of the superior temporal sulcus and right peri-calcarine regions had 15 poor-quality observations.

### 2.7. Blood Collection and DNA Extraction

Whole blood was obtained via venipuncture and immediately processed for biochemical analysis. The DNA extraction was conducted on whole blood using standard DNA isolation methods following the protocol described in the Qiagen QIAamp DNA Blood Mini Kit Qaigen, Hilden, Germany [32] and DNA concentration was analysed and measured using Thermo Scientific NanoDrop Thermo Scientific, Wilmington, DE, USA 2000/2000c spectrophotometers. A nucleic acid concentration > 10 ng/µL and 260/280 ratio (1.86–2.0) were considered normal [32].

### 2.8. APOE Analysis

Purified polymerase chase reaction (PCR) products obtained through DNA extraction in were sequenced by Gehrmann Laboratories, Research Road, University of Queensland, QL, AU. Single-nucleotide polymorphism (SNP) genotyping was performed at the Australian Genome Research Facility (AGRF) Brisbane Node at the University of Queensland using Agena Bioscience MassARRAY^®^, Agena Bioscience, San Diego, CA, USA service on a MA4 mass spectrometer and iPLEX GOLD chemistry. Haplotypes corresponding to *APOE* ε2, ε3 and ε4 were then deduced. Participants were classified corresponding to the presence of rs429358, rs7412 carrier status based on the presence of an ε4 allele. Genotyping was conducted on all 109 participants.

### 2.9. Statistical Analysis

Statistical analysis and graph generation was performed using SPSS software version 26 for Mac (SPSS, Inc., Chicago, IL, USA). The association between APOE ε4 allele carriage and changes in various measurable parameters was examined using the generalised estimating equation (GEE). The GEE model was adjusted with age as a covariate, gender as a cofactor, and left or right eye as within-subject variability. Predicted Wechsler Adult Intelligence Scale (WAIS-IV) full-scale IQ was used as a covariate for all neuropsychological test scores and estimated total intracranial volume (eTIV) as a covariate for all MRI parameters. *p* values of <0.05 were considered statistically significant.

## 3. Results

A total of 109 aging subjects were included in the final analysis. Table 1 shows the demographic characteristics of all included subjects. All participants were identified as cognitively healthy participants and had an MMSE score of >25 [33]. Mean age (SD) at the entry of the study was 67.1 (9.0) and 51% of the cohort was female. Seventy percent of the study population were APOE ε3/ε3 homozygous, 22% carried at least one ε4 allele (20.2% heterozygote, 1.8% homozygous) and 13% were either ε2/ε2 (0.9%) or ε2/ε3 (12.8%). There were no significant differences between APOE groups in gender (χ^2^ = 2.57, *p* = 0.109) or age (*p* = 0.680) distribution.

### 3.1. mfVEP and RNFL Thickness Analysis

Assessing the relationship between sectoral regions and global RNFL thickness showed no significant differences apart from the temporal RNFL (*p* = 0.001), in which APOE ε4 carriers had thinner RNFL compared to non-carriers, representative of a less healthy retina. Analysis of the posterior pole revealed no observable difference between the two groups. (see Table 2). The mfVEP amplitude and latency showed no APOE group differences within the study population (Table 3).

### 3.2. Optical Coherence Tomography–Angiography Analysis (OCT-A)

There were significant differences in the FAZ area of the SCP (*p* < 0.001) and the FAZ area of the DCP (*p* = 0.003) between the two groups. Contrary to our hypotheses, the FAZ appeared healthier (smaller avascular area) in carriers; here, a reduced FAZ is beneficial, as a larger FAZ is seen in disease. Regarding circularity and VD, the greater the score the better. APOE ε4 carriers had higher VD of both the SCP (*p* = 0.592) and DCP (*p* = 0.770) than non-carriers, consistent with better retinal vascular health. The APOE ε4 carriers also had larger FAZ circularity of the DCP than non-carriers (*p* = 0.034), while there was no difference in the FAZ circularity of the SCP (*p* = 0.111). The FAZ perimeter was higher, meaning a larger FAZ in both the superficial capillary plexus (SCP) (*p* = 0.003) and the deep capillary plexus (DCP) (*p* = 0.004) in non-carriers than carriers.

### 3.3. Neuropsychological Test Scores Analysis

Twenty-four cognitive parameters across various cognitive domains were analysed for association with APOE carriage. GEE models assessing relationships between neuropsychological test scores and APOE genotype status, adjusted for age, sex and predicted IQ showed no relationships for most of the tests except logical memory (immediate) (*p* = 0.014 *), where the score was higher in participants who were carriers (see Table 3).

**Table 3:** Differences between APOE non carriers and carriers for various domains (cognitive, mfVEP and MRI brain volumes).

Carrier Status	APOE ε4− (n = 85)	APOE ε4+ (n = 24)	B-Value	95% CI	*p*-Value
	Mean	Mean			
Cognitive Test (Score)					
Predicted WAIS-IV_FSIQ	112	113.3	−1.47	−4.906 to 1.967	0.402
LM Immediate	12.6	14.8	−2.246	−4.030 to −0.461	0.014 *
LM Delay	11.3	12.8	−1.447	−3.524 to 0.631	0.172
LM Recognition	12	12.2	−0.22	−1.009 to 0.570	0.586
CVLT_TL	41.3	45	−3.547	−8.980 to 1.886	0.201
CVLT sdfr	8.8	9.6	−0.585	−2.282 to 1.112	0.499
CVLT ldfr	9.3	9.8	−0.273	−2.101 to 1.554	0.769
Letter Fluency	42.2	44.4	−1.817	−6.948 to 3.314	0.488
Category Fluency	19.7	19.5	0.147	−1.679 to 1.972	0.875
SDMT	48.5	48.9	−0.283	−4.653 to 4.087	0.899
DSF	11	10.9	0.169	−0.847 to 1.185	0.744
DSB	9.2	8.4	0.905	−0.038 to 1.848	0.06
DS Seq	8	8.4	−0.348	−1.055 to 0.358	0.334
DS Total	28.2	27.6	0.726	−1.265 to 2.717	0.475
TMA	32.6	33.4	−0.277	−4.030 to 3.476	0.885
TMB	79	92.7	−11.14	−34.891 to 12.610	0.358
BNT_NCS	27.4	27.7	−0.414	−1.468 to 0.640	0.441
MMSE	28.4	28.7	−0.363	−1.099 to 0.372	0.333
GDS	1.9	1.4	0.504	−1.062 to 0.923	0.288
RCFT_1	33.7	34.2	−0.619	−1.962 to 0.723	0.366
RCFT_2	17.3	19.2	−1.97	−5.351 to 1.411	0.253
RCFT_3	16.6	17.5	−0.813	−4.600 to 2.973	0.674
Visual Evoked Potential					
VEP Amplitude (nV)	141	154.1	−5.601	−29.539 to 18.337	0.647
VEP Latency (ms)	143.6	139.4	4.241	−2.981 to 11.462	0.25
MRI Brain Volumes (mL)					
ETICV	1,567,758	1,548,422	−39,335.344	−112,315.810 to 33,645.121	0.291
TGMV	600,412	597,664	−14,160.752	−40,569.022 to 12,247.518	0.293
Cortex Volume	438,318	437,714	−12,689.852	−34,193.327 to 8813.623	0.247
CWMV	447,808	444,803	−5479.851	−20,893.032 to 9933.330	0.486
GM Cingulate	18,396	17,873	−180.946	−1182.660 to 820.768	0.723
GM Parietal	102,065	100,950	−1798.391	−7333.500 to 3736.717	0.524
Entorhinal Volume	3798	3840	−132.203	−472.440 to 208.034	0.446
Third Ventricle	1534	1552	−148.685	−400.656 to 103.286	0.247
Forth Ventricle	1970	1796	116.368	−68.410 to 301.146	0.217
Thalamus	13,368	13,225	−127.904	−810.665 to 554.857	0.713
Putamen	9110	8935	3.211	−748.604 to 755.026	0.993
Hippocampus	7962	7795	69.839	−333.016 to 472.694	0.734

Abbreviations: RNFL = retinal nerve fibre layer; mfVEP = multifocal visual evoked potential; Amp = amplitude; Lat = latency; nV = nanovolts; ms = milliseconds; GM = grey matter; LM = logical memory; CVLT = California Verbal Learning Test; TL = total learning; sdfr = short-delay free recall; ldfr = long-delay free recall; SDMT = Symbol Digit Modality Test; DS = digit span; TMT = Trail-Making Test; BNT_NCS = Boston Naming Test—no-cue score; RCFT = Rey Complex Figure Test; MMSE = Mini-Mental State Examination; GDS = Geriatric Depression Scale. ETICV = estimated total intracranial volume; TGMV = total grey matter volume; CWMV = cerebral white matter volume. * *p* < 0.05.

### 3.4. MRI Brain Volume Analysis

Analysis of selected regions of the brain that have been previously shown to play a role in aging and cognition were analysed for differences between APOE carriers and non-carriers. No significant differences were identified in these regions (Table 3).

## 4. Discussion

The APOE ε4 genotype is the strongest common genetic risk factor for sporadic AD. Understanding the relationship between APOE *ε*4 and various retinal parameters could provide novel therapeutic avenues and help with the early diagnosis of AD. In this study, we utilised OCT and OCT-A to analyse the retinal microvasculature and explore the morphological and characteristic features of the FAZ and retinal vasculature. We hypothesised that OCT-A parameters such as the FAZ and VD would be key indicators distinguishing APOE carrier status.

### 4.1. Our Findings

We found that several FAZ parameters were better in APOE carriers than non-carriers and that the differences were more pronounced in the deep layer of the FAZ. However, other imaging and testing modalities revealed no differences between APOE carriers and non-carriers. Performance of the participants in neuropsychological testing were similar, apart from performance in the immediate recall subtest of a story memory task. This is inconsistent with previous findings of APOE ε4 status associations with poorer performance on immediate and delayed memory tests [34]. However, these findings were in patients in the early stages of AD, unlike our cohort of aging participants, and only in patients with two ε4 alleles compared to those with one or zero ε4 alleles. Additionally, amongst the OCT parameters, only the temporal RNFL thickness was significantly reduced in APOE ε4 carriers compared to the non-carriers.

### 4.2. Comparisons to Previous Literature

Recent studies that have explored the retinal microvasculature in AD patients have found differences between the FAZ of healthy controls and clinically diagnosed AD patients [11,35], whilst others have found differences in both the superficial and deeper layers of the FAZ [36]. The APOE ε4 allele has also been associated with faster rates of GCIPL (ganglion cell inner plexiform layer) thinning in eyes of normal-tension glaucoma, further solidifying its potential influence across neurodegenerative diseases [37]. With our cohort being comprised of community-dwelling aging participants, the FAZ results should be interpreted with caution. Whilst our results show a decreased FAZ in the carrier cohort, the results are the opposite of what one would expect. We attribute this primarily to statistical chance and the nature of the cohort. The carrier cohort were classified as normal aging participants, with the *ε*4 allele expecting to show features of very early dysfunction. It may be possible that some individuals were at an early preclinical stage of early MCI (mild cognitive impairment). Another possible explanation found to explain this inverse association is the role of subretinal inflammatory changes. These changes, found in an AMD mouse model, have been shown to be isoform-dependent, with the APOE ε2 isoform a risk factor and the ε4 isoform protective. A growing number of studies have implicated a link between AD and retinal neurodegenerative disease that share several pathological events and risk factors. APOE ε4 is the strongest genetic risk factor for AD, whereas APOE ε2 is considered a protective factor; however, in retinal neurodegenerative diseases, paradoxically APOE ε4 is a protective factor and APOE ε2 is a risk factor [38]. Although there are many reports on the effects of APOE isoforms on AD initiation and progression, data about their role in retinal neurodegenerative diseases are limited and further studies are needed to reveal their differential effects on pathogenesis of these diseases. In our dataset, we only found one neuropsychological domain that showed a significant difference between APOE ε4 genotypes. A major risk factor for AD is the presence of the APOE ε4 allele; however, few studies have simultaneously investigated its role with respect to cognitive decline, specifically logical memory. Mormino et al. [39] reported that the presence of the APOE ε4 allele revealed greater decline in amyloid beta-positive/APOE ε4^+^ participants, with minimal decline in other groups. The results in our study are similar to those of Mormino et al. (2014), with APOE ε4^+^ carrier status showing a greater decline in logical memory immediate recall.

Again, contrary to expectations, we found no significant associations between the genotype groups with regard to MRI volumetric variables or mfVEP. The most probable explanation for this is that our cohort was comprised of aging participants, with little to no global alterations in the brain and visual pathway.

The association between RNFL thickness measurements and brain structural volumes is well known and established [40]. There is a direct correlation between RNFL thickness located in the temporal quadrant and hippocampal volume [41], with a reduced thickness of the retinal ganglion layer reported in both AD and mild-MCI subjects [42,43]. Although these associations have been reported previously, they offer the possibility for RNFL changes to be a potential early diagnostic marker for AD-related atrophy. The current study did not find any significant changes in the sectoral regions of the RNFL except for the temporal quadrant. Thinner RNFL, particularly in the temporal region, has previously been shown to correlate with older age in OCT analysis, especially in glaucoma [44,45,46]. It has also been reported as the most important nerve fibre layer thickness in MS (multiple sclerosis) estimates [47].The temporal RNFL supplies the macula. A loss of axons in the temporal quadrant may be due to associations of this region with central vision and consisting primarily of small parvocellular axons, which are more susceptible to damage. Changes in various RNFL sectors have been associated with disease progression and worsening cognitive decline, as well as a normal consequence of aging.

### 4.3. Strengths of Our Study

This study has several strengths, including the use of various neuroimaging techniques to measure retinal and brain parameters as well as cognitive performances in an aging cohort. Various techniques that could aid early detection but are not commonly used for AD disease progression clinically, such as OCT and OCTA, have been utilised. Eleven neuropsychological tests covering a range of cognitive domains, including those sensitive to hippocampal-dependent learning and memory impairments typically observed in AD. The SD-OCT assessments were performed on the same SD-OCT machine according to a standardised protocol. Moreover, the spectral domain technique provides the best quality and reproducibility available currently. The brain MRI measurements were also performed on the same machine and according to a standardised procedure.

### 4.4. Study Limitations

The following limitations should be considered. Firstly, a limitation was the relatively smaller sample, and this may have resulted in a lack of significant findings in some analyses, namely, the number of ε4ε4 participants on various analyses. Further investigations with large samples and longitudinal follow-up are required to validate any associations. Possible alterations to the methodology could include further subdividing the ε4ε4 cohort into carriers with two vs. one allele and assessing the correlation. Further studies should include AD and MCI participants to determine whether the evaluation of the retinal microvasculature using OCT-A has value as an early biomarker in AD. Access to amyloid PET imaging may be the next logical step to quantify and observe the downstream effects of the APOE ε4 allele in this study cohort. Finally, these analyses were conducted in a general cohort because the APOE ε4 allele frequency as well as its association with AD risk may be strongly influenced by ethnicity and geographic region. Comparisons among various ethnic groups would be interesting topics for future studies to better understand these complex associations [48].

## 5. Conclusions

This study provides valuable insights and builds on the existing literature available on the effects of the APOE ε4 allele and retinal measures in aging. This study illustrates that using various ophthalmic imaging modalities and neuropsychological testing may play a key role in the diagnosis of neurodegenerative conditions such as AD.

## Figures and Tables

**Table 1:** Demographic data of participants included in the study.

	Controls (n = 109)
Age, mean ± SD, years	61.1 ± 9.0
Sex (M/F), (n/n)	53/56
Male No. (%)	48.60%
Female No. (%)	51.4%
APOE ε4 carrier (n/%)	(24/22.3%)
APOE ε4 non-carrier (n/%)	(85/77.9%)
Education Level (n)	
Missing	8
Secondary School	15
Bachelor or TAFE	70
Master’s	11
PhD	7
General Health Status (n/% of Cohort)	
Diabetes Mellitus	5/4.6%
Hypertension	39/36%
Hypercholesterolemia	40/36.6%
Systolic Blood Pressure, mm Hg mean—(SD)	132.1 ± 15.2
Diastolic Blood Pressure, mm Hg mean—(SD)	85.9 ± 9.1
VA, mean ± SD, LogMAR	0.03 ± 0.05
MMSE, mean ± SD	28.42 ± 2.

Abbreviations: VA = visual acuity; logMAR = logarithm of the minimum angle of resolution; MMSE = Mini-Mental State Examination.

**Table 2:** Optical coherence tomography and optical coherence tomography–angiography parameters of APOE ε4 carriers and non-carriers.

	APOE ε4−	APOE ε4+	*p*-Value
N	85	24	
Age, mean ± SD, years	67.01 (9.1)	67.6 (8.9)	0.68
Sex (M/F) (n/n)	43/42	9/15	0.11
Retinal Microvasculature, mean			
FAZ area (mm^2^) SCP	0.40 (0.2)	0.30 (0.1)	<0.001 *
FAZ area (mm^2^) DCP	0.39 (0.2)	0.29 (0.1)	0.003 *
FAZ perimeter (mm)—SCP	2.31 (0.5)	1.90 (0.2)	0.001 *
FAZ perimeter (mm)—DCP	2.32 (0.4)	2.01 (0.1)	0.004 *
FAZ circularity index—SCP	0.91 (0.1)	0.90 (0.0)	0.111
FAZ circularity index—DCP	0.89 (0.01)	0.92 (0.02)	0.034 *
VD (%)—SCP	23.07 (5.5)	24.59 (5.2)	0.592
VD (%)—DCP	20.82 (8.0)	21.71 (3.3)	0.771
Retinal layer thickness, (μm) ± SD			
RNFL G	96.5 (9.7)	96.0 (7.6)	0.698
RNFL T	71.7 (12.3)	66.7 (8.3)	0.01 *
RNFL TS	130.8 (20.9)	130 (21.4)	0.758
RNFL TI	145.2 (20.6)	145.6 (20.4)	0.904
RNFL N	77.3 (12.1)	76.6 (12.5)	0.857
RNFL NS	108.1 (21.4)	105.3 (22.1)	0.641
RNFL NI	110.9 (22.3)	117.1 (19.5)	0.229
RNFL S	119.5 (14.8)	118.9 (13.5)	0.787
RNFL I	128.1 (18.4)	131.8 (16.8)	0.462
P Pole T	66.7 (5.2)	66.3 (6.0)	0.823
P Pole TS	67.5 (5.5)	65.6 (6.1)	0.227
P Pole TI	67.1 (5.8)	65.4 (5.8)	0.202

Abbreviations: Abbreviations: RNFL = retinal nerve fibre layer; G = global; T = temporal; S = superior; TS = temporal superior; TI = temporal inferior; N = nasal; NS = nasal superior; NI = nasal inferior; I = inferior; P Pole = posterior pole; FAZ = foveal avascular zone; SCP = superior capillary plexus; DCP = deep capillary plexus; VD = vessel density; peri = perimetry; cir = circularity. * *p* < 0.05.

## Data Availability

The data presented in this study are available on request from the corresponding author. The data are not publicly available due to patient confidentiality.
